# The use of fully covered self-expandable metal stents in the endoscopic treatment of portal cavernoma cholangiopathy

**DOI:** 10.1186/s12876-023-03042-5

**Published:** 2023-11-28

**Authors:** Abdullah Murat Buyruk, Çağdaş Erdoğan, Fatih Tekin, İlker Turan, Ömer Özütemiz, Galip Ersöz¹

**Affiliations:** 1 Department of Gastroenterology, Ege University School of Medicine, İzmir, Turkey; 2 Department of Gastroenterology, University of Health Sciences, Ankara Etlik City Hospital, Ankara, Turkey

**Keywords:** Portal cavernoma cholangiopathy, Portal hypertensive biliopathy, Endoscopic treatment, FCSEMS, Biliary strictures

## Abstract

**Background and aims:**

There are different therapeutic approaches for biliary strictures and reducing portal hypertension in patients with symptomatic portal cavernoma cholangiopathy (PCC). Endoscopic treatment includes endoscopic biliary sphincterotomy (EST), dilation of stricture with a biliary balloon, placement of plastic stent(s) and stone extraction. Fully covered self-expandable metal stent (FCSEMS) is placed as a rescuer in case of haemobilia seen after EST, dilation of stricture and removal of plastic stent rather than the stricture treatment itself. In this retrospective observational study, we sought to assess the clinical outcomes of FCSEMS as the initial treatment for PCC-related biliary strictures.

**Materials and methods:**

Twelve symptomatic patients with PCC both clinically and radiologically between July 2009 and February 2019 were examined. Magnetic resonance cholangiopancreatography (MRCP) and cholangiography were employed as the diagnostic imaging methods. Chandra–Sarin classification was used to distinguish between biliary abnormalities in terms of localization. Llop classification was used to group biliary abnormalities associated with PCC. Endoscopic partial sphincterotomy was performed in all the patients. If patients with dominant strictures 6-8-mm balloon dilation was first performed. This was followed by removal of the stones if exist. Finally, FCSEMS placed. The stents were removed 6–12 weeks later.

**Results:**

The mean age of the patients was 40.9 ± 10.3 years, and 91.6% of the patients were male. Majority of the patients (n = 9) were noncirrhotic. Endoscopic retrograde cholangiopancreatography (ERCP) findings showed that 11 of the 12 patients were Chandra Type I and one was Chandra Type IIIa. All the 12 patients were Llop Grade 3. All patients had biliary involvement in the form of strictures. Stent placement was successful in all patients. FCSEMSs were retained for a median period of 45 days (30–60). Seven (58.3%) patients developed acute cholecystitis. There was no occurrence of bleeding or other complications associated with FCSEMS replacement or removal. All patients were asymptomatic during median 3 years (1–10) follow up period.

**Conclusions:**

FCSEMS placement is an effective method in biliary strictures in case of PCC. Acute cholecystitis is encountered frequently after FCSEMS, but majority of patients respond to the medical treatment. Patients should be followed in terms of the relapse of biliary strictures.

## Introduction

Portal cavernoma cholangiopathy (PCC) refers to the extrahepatic biliary system abnormalities observed in patients with portal cavernoma [1]. There are various names (“portal hypertensive biliopathy” [2], “portal cavernoma associated cholangiopathy” [3], “portal cavernoma cholangiopathy” [4], “cholangiopathy associated with portal hypertension” [5], “pseudosclerosing cholangitis” [6] and “pseudocholangiocarcinoma” [7]) for this disease in the literature.

Direct pressure on the extrahepatic bile ducts exerted by the mass of a thrombus located in the portal vein as well as the pressure on the bile ducts exerted by the dilation of venous structures because of venous drainage impairment in the extrahepatic bile duct after thrombosis are considered the primary causes of PCC development [8, 9]. In addition, ischemia, which develops because of arterial flow deterioration indirectly affected by portal vein thrombosis extension to the veins that drain the biliary system, is thought to have an impact on the pathogenesis by causing short-segment fibrotic strictures and angulations in the extrahepatic bile ducts [10, 11].

In radiological examinations, PCC findings of varying severities were observed in a vast majority (80–100%) of patients with extrahepatic chronic portal vein thrombosis [12–16]. While most of these patients were asymptomatic, 21% (5–50%) were found to exhibit symptoms. Risk factors for development of symptomatic PCC include older age of the patient, long duration of extrahepatic portal vein obstruction, dilated segments of bile ducts, and the presence of gallstones and bile duct stones [13, 14]. Jaundice, abdominal pain, and fever are the most common symptoms observed in symptomatic patients. Laboratory and imaging methods are considerably helpful in diagnosing these patients. Laboratory tests can show elevated alkaline phosphatase (ALP), gamma-glutamyl transferase (GGT), and bilirubin levels (predominantly direct bilirubin) consistent with cholestasis. Imaging can reveal enlargement of the intrahepatic bile ducts (IHBDs) and extrahepatic bile ducts (EHBDs), external pressure on the EHBDs, strictures and wall thickening in the bile duct, angulation, and displacement of the EHBD, cholelithiasis, choledocholithiasis, hepatolithiasis, and hilar masses suggestive of malignancy [6, 7, 17, 18].

Treatment is not recommended for asymptomatic patients with PCC. The prognosis of patients with symptomatic and severe cholestasis is poor if an effective treatment is not administered. These patients may experience recurrent cholangitis, progressive liver dysfunction, and secondary biliary cirrhosis and may need liver transplantation [19]. Both the bile ducts and portal venous system should be considered for the treatment of patients with symptomatic PCC. Endoscopic treatment is the first-line treatment, and its methods include endoscopic sphincterotomy, balloon dilation, stone extraction, nasobiliary catheter drainage, and plastic stent placement. Due to the high rate of relapse after endoscopic treatment, repeated interventions may be required to perform bile duct drainage [19–22]. This leads to the development of cholangitis episodes caused by resistant nosocomial infections. In addition, the rate of unpleasant complications such as haemobilia is high in patients undergoing plastic stent replacement. Fully covered self-expandable metal stent (FCSEMS) placement is performed as a rescue procedure in case of haemobilia after endoscopic sphincterotomy (EST), dilation of stricture, and removal of plastic stent rather than for the treatment of stricture itself [23].

In this study, we sought to assess the clinical outcomes of FCSEMS as the initial treatment for PCC-related biliary strictures.

## Materials and methods

**Fig. 1:**
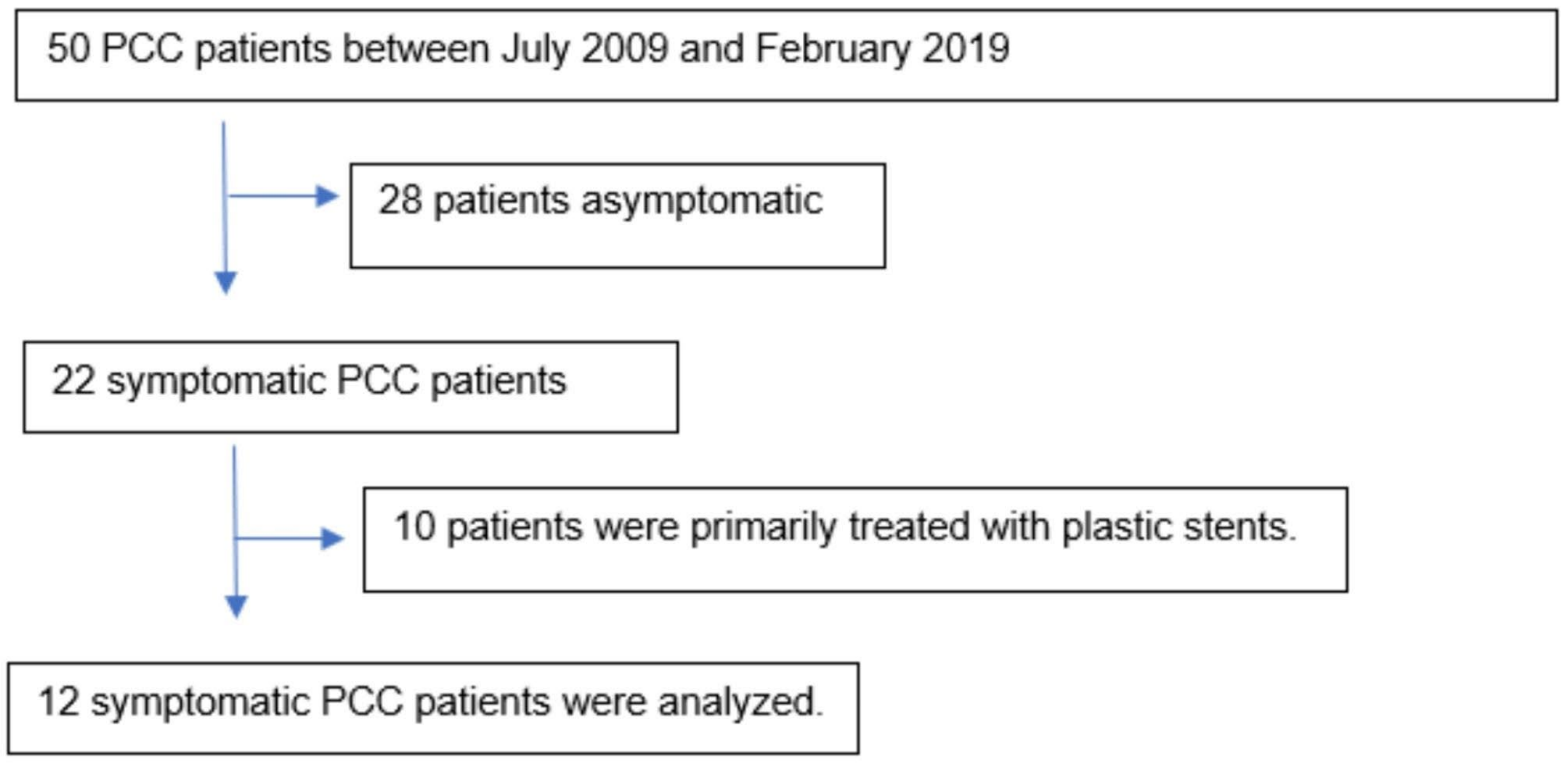
Study Flow Chart

Twelve symptomatic patients who had been diagnosed with PCC both clinically and radiologically between July 2009 and February 2019 were included in the study. Flow chart of the study is given in Fig. 1. Symptoms identified to be associated with PCC were abdominal pain, jaundice, itching, and fever. Magnetic resonance cholangiopancreatography (MRCP) and cholangiography were employed as the diagnostic imaging methods. Additionally contrast-enhanced CT and ultrasound Doppler studies were performed on all patients. This information has been added to the article. The main alterations shown by MRCP and/or cholangiography were bile duct stenosis, angulations, intra and extrahepatic dilations, parietal irregularities, bile duct angulations, choledochal varices, and intra and extrahepatic lithiasis. Chandra–Sarin classification was used to distinguish between biliary abnormalities in terms of localization [14]. According to the Chandra–Sarin classification, Type I, II, IIIa, and IIIb refers to the involvement of extrahepatic bile duct alone, intrahepatic bile duct alone, extrahepatic, and unilateral IHBDs (left or right), and extrahepatic and bilateral IHBDs, respectively. Llop classification was used to group biliary abnormalities associated with PCC [24]. Grade I was used to describe any abnormalities or angulations in the biliary tree, Grade II was used to describe indentations or strictures without dilatation, and Grade III was used to describe strictures with dilation (defined as intrahepatic duct 4 mm or extrahepatic duct 7 mm). Patients with primary sclerosing cholangitis, bile duct neoplasms, biliary tract surgery, acquired immunodeficiency syndrome cholangiopathy, biliary parasitosis, congenital abnormalities of the biliary tract, ischemic bile duct stricture, toxic bile duct strictures, strictures due to autoimmune, chronic pancreatitis and IgG4 cholangiopathy were excluded from the study.

Endoscopic partial sphincterotomy was performed in all the patients. If patients with dominant strictures in the common hepatic and common bile ducts also had a stone in either of the ducts, 6- or 8-mm balloon dilation was first performed at a location distal to the stone on the dominant stricture according to the diameter of the duct. This was followed by removal of the stones with a stone extraction balloon and finally FCSEMS placed. If there were no stones in patients with dominant strictures in the common hepatic and common bile ducts, only FCSEMS placement was performed. In patients who had stones in the IHBD, only a metallic stent was placed just below the bifurcation during the first session. The stones in the IHBD were removed after metallic stent removal and dilation at the base of the common duct with a 6- or 8-mm balloon. In patients with PCC who had involvement of the bifurcation and the right-left hepatic ducts, the stent was expanded in the right or left hepatic duct and then pulled back and placed right below the bifurcation. The length of the stent was determined by calculating the distance between the proximal end of the dominant stricture and the papilla. FCSEMSs of 6 or 8 or 10 cm-length and 10 mm-diameter were placed in all patients. The stent was removed 6–12 weeks later. Initially, the stents were removed at 6 weeks until two patients experienced recurrence. Consequently, we decided to remove the stents at 12 weeks in the remaining 10 patients. Clinical, laboratory, and radiological improvements were determined in all patients following a 6–12-week FCSEMS treatment.

Antibiotic therapy (cefotaxime in six patients and ciprofloxacin in five patients) was administered in a single dose 30 min before and for at least 5 days after all the procedures. Patients were monitored in the ward for 1 day after the procedure. All patients were administered ursodeoxycholic acid (15 mg/kg) as maintenance therapy.

## Results

**Fig. 2:**
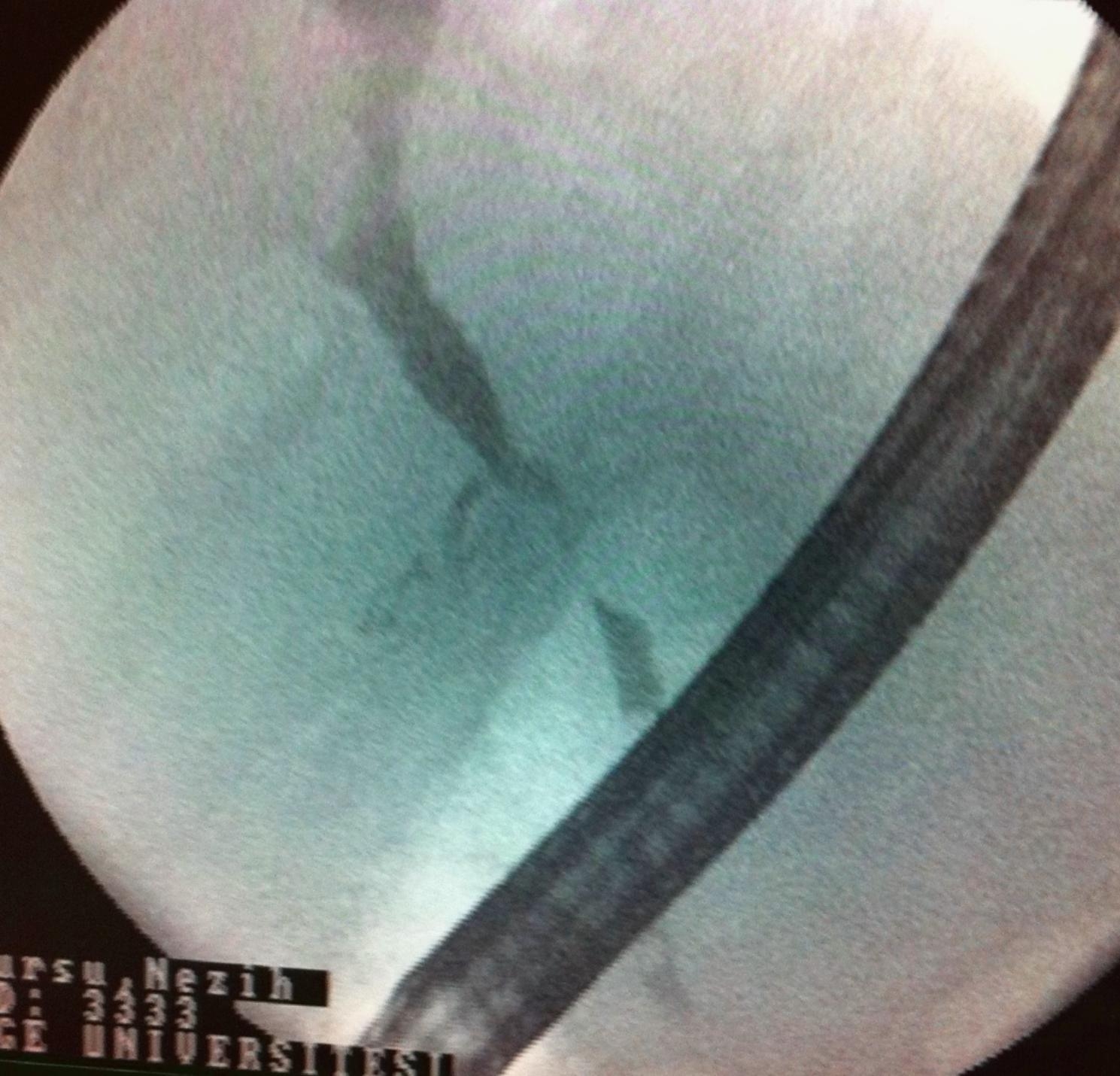
Biliary dominant stricture of PCC

**Fig. 3:**
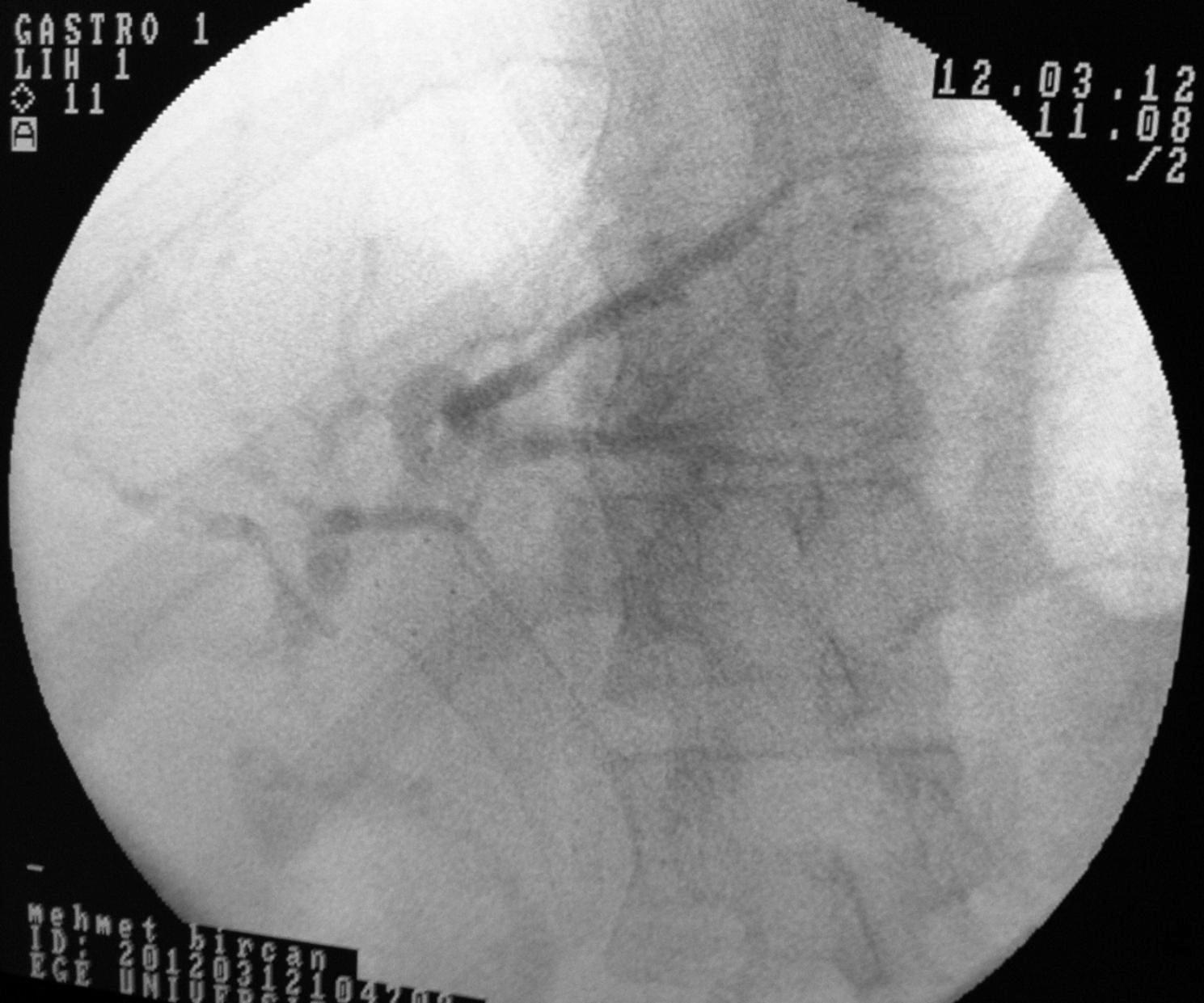
FCSEMS placement

**Fig. 4:**
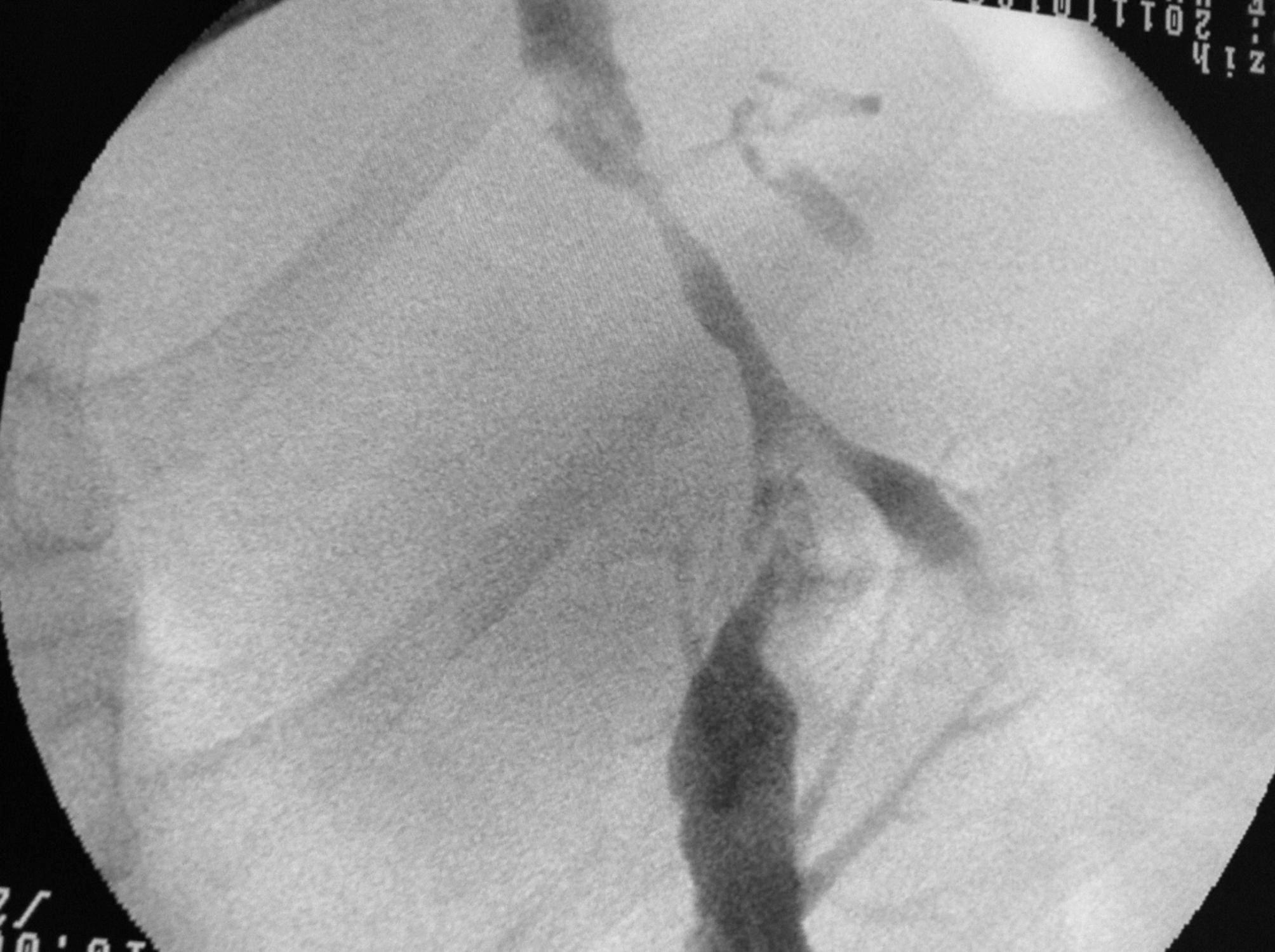
Improvement of stenosis after FCSEMS application

Twelve patients with symptomatic PCC who underwent FCSEMS as primary endoscopic treatment between July 2009 and February 2019 were included in the study. The mean age of the patients was 40.9 ± 10.3 years, and 91.6% of the patients were male. In terms of symptoms, all 12 patients complained of abdominal pain and/or jaundice. Laboratory investigations revealed elevated bilirubin (5.9 µmol/L; reference < 1.0 µmol/L), alkaline phosphatase (328 U/L; reference < 120 U/L), γ-glutamyl transpeptidase (135 U/L; reference < 38 U/L). Majority of the patients (n = 9) were noncirrhotic. One of the noncirrhotic patients had a cystobiliary fistula and portal vein thrombosis secondary to a hydatid cyst. None of the patients had inherited coagulation disorders that may have caused portal vein thrombosis, and none of them received formal anticoagulation. Regarding the etiology in cirrhotic patients, one patient had hepatitis B virus infection and another patient had autoimmune hepatitis. The remaining patients were considered to have cryptogenic cirrhosis. Pre-procedural demographic data were given in Table 1. Three patients had undergone splenectomy for an unknown cause. Endoscopic retrograde cholangiopancreatography (ERCP) findings showed that 11 of the 12 patients were Chandra Type I and one was Chandra Type IIIa. According to the classification system proposed by Llop et al. [24], all the 12 patients belonged to Grade 3. As per ERCP findings, all patients had biliary involvement in the form of strictures. The strictures involved the common bile and common hepatic ducts in all patients, except two in whom the strictures involved the left intrahepatic duct. Choledocholithiasis was observed in four patients and hepatolithiasis was observed in two. Stent placement was successful in all patients. FCSEMSs were retained for a median period of 45 days (range: 30–60 days). After stent placement, eight patients developed post-ERCP abdominal pain without the presence of pancreatitis; all of them were treated with intravenous administration of 0.5 mg of hydromorphone. Seven (58.3%) patients developed acute cholecystitis, and abdominal pain associated with acute cholecystitis was observed on day 2 after ERCP in all of them. Percutaneous cholecystostomy was performed in two of these patients, whereas the remaining five were received conventional medical treatment. In the group that developed acute cholecystitis, the length of hospital stay was significantly higher compared to the group that did not exhibit cholecystitis (9 days vs. 3 days, p = 0.04). Perioperative bleeding was observed during EST in two patients and during portal vein cannulation in one patient. Fortunately, the bleeding stopped after FCSEMS placement, and none of the patients experienced bleeding recurrence. The metallic stent had migrated into the common bile duct in one patient who was scheduled to undergo stent removal. In this patient, the stent was easily removed using a stent extractor. A downside in our study was that a second FCSEMS placement was performed due to the recurrence of upper right abdominal pain and jaundice complaints and detection of biliary strictures in the same location 6 years later in one patient and 2 years later in another. There was no occurrence of bleeding or other complications associated with FCSEMS replacement or removal. At the time of writing this article, all patients were asymptomatic [median follow-up: 3 years (range 1–10 years)]. Endoscopic treatment data were given in Table 2. Figures 2, 3 and 4 show the application of FCSEMS to the dominant stricture of one of the study participants before, during, and after the application.

**Table 1:** Pre-procedure demographic data

Age (years)	40.9 (28–54)
**Gender (n)**	
Male Female	11 1
**Etiology (n)**	
Cirrhosis ♣ HBV ♣ Autoimmune hepatitis ♣ Cryptogenic cirrhosis Non-cirrhotic portal hypertension	3 1 1 1 9
**Symptom (n)**	
Jaundice Abdominal pain Nausea Vomiting Fever	9 8 4 4 3

**Table 2:** Data on endoscopic treatment

Parameters	n = 12
**Cholangiography findings (n)** Strictures	
♣ Common bile duct ♣ Common hepatic duct ♣ Left intra-hepatic ducts ♣ Right intra-hepatic ducts	12 12 2 0
Gallstone(s)	
♣ Choledocholithiasis ♣ Hepatolithiasis	4 2
**Time to stent removal**	
6 weeks 12 weeks	8 4
**Balloon dilatation**	6
**Removal of biliary stones**	6
**Success rate of FCSEMS placement**	100%
**ALP before initial ERC**	328 (420 − 229) U/L
**ALP after SEMS placement**	128 (144 − 112) U/L
**Total bilirubin before initial ERC**	5.9 (8.4–3.6) mg/dL
**Total bilirubin after SEMS placement**	1.2(2.1–0.9) mg/dL
**Complication (n)**	
Post-ERC abdominal pain Acute cholecystitis • Percutaneous cholecystostomy • Medical treatment Bleeding • Portal vein cannulation	8 7 2 5 1 1
**Recurrence (n)**	2

## Discussion

In this single-center retrospective observational study, the efficacy of FCSEMS placement was investigated in patients with symptomatic PCC. Accordingly, FCSEMS was shown to be highly effective in patients with PCC. While the symptoms and strictures were eliminated in all patients with this treatment method, the most common complication observed was acute cholecystitis. The clinical presentation of acute cholecystitis was monitored in all the patients for the first 48 h; majority of the patients responded to conservative treatment. Therefore, according to the results of the present study, we recommend to closely monitor in patients with PCC with gallbladder who are planned to undergo FCSEMS placement, especially during the first 48 h.

Cholangiographic changes (narrowing, segmental upstream dilatation irregularity, undulation, nodular extrinsic defects, and gallstones) can be observed in almost all patients with PCC [12–16]. Abnormalities were seen in the common bile duct, left intra-hepatic ducts, and right intra-hepatic ducts [16]. In general, the main problem underlying the symptoms in patients with symptomatic PCC is thought to be the strictures caused by varices and fibrosis at the common hepatic duct level below the bifurcation, stones in the common bile and hepatic ducts, and in rare cases, stones in the IHBD [9, 14]. In this study, information regarding the cholangiography findings of all patients with PCC could not be provided since the study only included patients with symptomatic PCC. The most common cholangiography findings in our patients were strictures in the common bile duct, common hepatic duct, and/or left hepatic duct. In addition, half of the patients had stones in the common bile duct and/or IHBD.

In patients with symptomatic PCC, endoscopic sphincterotomy, balloon dilation, plastic stent placement, and nasobiliary drainage were the most employed endoscopic treatment methods [19–21, 25]. There are a few studies in the literature that included a limited number of patients receiving endoscopic treatment for PCC. The study with the largest number of patients with PCC who received endoscopic treatment was that of Saraswat et al. [26],; they published the data of 130 ERCP procedures performed for biliary strictures in 20 symptomatic patients. In this study, endoscopic sphincterotomy with stone extraction (SE) was performed in eight patients with common bile duct stones and nine patients were treated with plastic stent placement. Eleven patients with postoperative benign biliary strictures were treated (in a total of 101 procedures) with balloon dilation and plastic stent placement; exchange was performed every 3–4 months until liver function tests returned to normal; additionally, cholangiograms returned to normal in 8 of the 11 patients. Complications of ERCP included haemobilia in 9 of 130 and cholangitis in 40 of 130 procedures. As reported in this study, the most common complication (6–60%) observed in the endoscopic treatment of PCC was cholangitis [19, 23, 26]. Cholangitis is caused by an incomplete clearance of calculi and debris accumulated above the strictures. In addition, blockage of plastic stents occurring within a short period of time due to frequent haemobilia is considered another factor involved in the etiology of cholangitis. Consequently, these patients required repeated plastic stent replacements. Inadequate patient adherence to stent replacement time is also thought to cause cholangitis episodes [26]. According to previous studies, the need for repeated endoscopic interventions and the high complication rates associated with it led to a need for alternative treatments for PCC.

There is scarcity of data on the use of FCSEMS in patients with PCC [21, 23, 27–30]. Reports of previous studies are summarized in Table 3. The use of FCSEMSs including in the common hepatic duct and the common bile duct is thought to obliterate the varices on the surface of the bile duct by compressing the said varices and dilate the fibrotic strictures that can also be encountered in such patients much more effectively [27]. FCSEMS is not a common practice that has been a part of routine practice in the endoscopic treatment of patients with symptomatic PCC, and it is usually employed as a rescue treatment or in patients with a short life expectancy in the form of uncovered metallic stents [21, 23, 27–30]. A group of researchers used uncovered metallic stents in three patients, which we believe was already contraindicated for this treatment [27]. Another researcher published a case presentation and mentioned using a covered metallic stent, wherein the patient had a covered stent replacement due to bleeding that occurred while removing the previous stent and did not have to undergo stent replacement again [29]. Another researcher had to remove the stent because the patient who had a biliary stricture associated with PCC developed empyema of the gallbladder but reported that the stricture had improved [28]. This study is of considerable importance as it reported the highest number of symptomatic patients with biliary strictures associated with PCC undergoing FCSEMS placement in the literature. In this case series, cholangitis episodes were almost never encountered in patients treated with FCSEMS compared to those treated with other endoscopic methods. The relapse rate was very low (18.1%) and success was achieved with long-term FCSEMS application in all patients who had recurrence. Therefore, FCSEMS indwell time was increased to 12 weeks in the last seven patients.

**Table 3:** Reports of previous studies

Reference	Number of Patients	Biliary Findings	Treatment	Follow up	Outcome
Perlemuter et al. [35], 1996	8	CBD stenosis CBD stones (2)	ES + PS + NBD UDCA	Multiple ERCPs	4 resolutions 2 deaths 2 relapses
Condat et al. [3], 2003	7	CBD stenosis	ES + PS UDCA	Multiple ERCPs	4 resolutions 2 relapses 1 haemobilia
Sezgin et al. [25], 2003	10	CBD stenosis	ES + PS + NBD	Multiple ERCPs	5 cholangitis 1 haemobilia 1 death
Khare et al. [40], 2005	13	CBD stenosis	ES + PS + BD	Multiple ERCPs	1 death 5 relapses
Dhiman et al. [2], 2007	12	CBD stenosis CBD stones (5)	PSS ES + PS	Multiple ERCPs	2 cholangitis 5 relapses
Vibert et al. [41], 2007	19	CBD stenosis CBD stones (4)	ES + PS	Multiple ERCPs	17 resolutions 3 deaths
Oo et al. [23], 2009	13	CBD stenosis CBD stones (10)	ES + PS UDCA	Multiple ERCPs 1 FCSEMS	2 haemobilia 3 sepsis
Saraswat et al. [26], 2014	20	CBD stenosis	ES + PS	Multiple ERCPs	40 cholangitis 9 haemobilia
Goenka et al. [30], 2014	1	CBD stenosis	FCSEMS (for hemobilia)	Resolution	resolution

CBD: common bile duct; ES: endoscopic sphincterotomy; PS: plastic stent; NBD: nasobiliary drainage; BD: balloon dilation; UDCA: ursodeoxycholic acid; ERCP: endoscopic retrograde cholangiopancreatography

Patients undergoing endoscopic treatment for PCC are at high risk of developing haemobilia [20, 29, 30]. Bleeding can be monitored during endoscopic sphincterotomy, balloon dilation, and stent replacement [20, 29, 31]. Distinguishing between stones and varices is highly challenging in cases wherein bile duct-related defects are observed on cholangiogram. Severe bleeding due to varices can be encountered during the elimination of these defects using a SE balloon or Dormia basket and may even lead to disrupted duodenoscopic view, which makes it more difficult to continue the procedure [31]. During the endoscopic treatment of our patients with PCC, the guide placed in the IHBD was kept in place until the end of the procedure when removing the stones in the bile duct with a balloon, and even if there was severe bleeding after removal, leading to disrupted endoscopic view, bleeding could be stopped, and effective bile duct drainage was achieved by placing a covered metallic stent under endoscopic guidance. As a matter of fact, one of our patients experienced severe bleeding during the repositioning of the guide that was dropped during sphincterotome-aided SE since the guide had entered the portal vein branches. The bleeding could be stopped after an FCSEMS was placed during the same session following the insertion of a guide into the IHBD under endoscopic guidance. In addition, venous collaterals in the region of the ampulla of Vater may lead to bleeding during papillotomy [27]. Covered metallic stents are also effective in stopping postpapillotomy bleeding [32]. Mutignani et al. [27], reported regarding perioperative bleeding that took place while removing stents in three patients who underwent plastic stent placement for PCC. In the said study, bleeding was stopped by using a covered metallic stent in the first patient (closure of the bilioportal fistula). In previous studies, a pigtail stent was most commonly used during endotherapy. It was stated that pigtails stent were preferred over straight stents as they carry increased bleeding risk as a result of their flank angles [27]. In our patients, no remarkable bleeding was observed after stent replacement.

Partially covered metallic stents are not recommended for patients with PCC. This is because “intimal hyperplastic tissue within metallic stents” are present on the uncovered areas located at the ends of partially covered stents, which can lead stent obstruction as well as bleeding and difficulties during stent removal [27, 29].

Although endoscopic treatment can temporarily relieve bile duct obstruction, it does not treat the underlying cause in patients with PCC. Therefore, it cannot be expected to be effective in the long term. On the other hand, performing direct surgery on the bile duct is not recommended as the first-line treatment due to high mortality and morbidity risks associated with it [33]. In this respect, shunt surgery is the recommended treatment option [33, 34]. It enables the decompression of varices that cause bile duct obstruction, thereby treating the stricture in the bile duct as well as leading to decreased risk of variceal bleeding due to the drop in portal pressure [32, 33]. It also facilitates performing surgery on the bile duct or endoscopic treatment that may be required after shunt surgery. Although shunt surgery was reported to have a low risk of morbidity and mortality, the most important disadvantage of the procedure is its invasive nature [34]. We believe that the use of FCSEMS may lead to a decreased need for proximal splenorenal shunt surgery since majority of our patients did not experience recurrence in the long term. To further validate this, prospective studies including many patients are necessary.

In this study, all patients were continuously administered ursodeoxycholic acid (UDCA) in order to prevent stricture recurrence and stone formation. However, UDCA administration has a limited role in PCC treatment. Several authors have reported the concomitant use of UDCA in patients with symptomatic PCC undergoing endoscopic treatment [3, 23, 24, 35]. Perlemuter et al. [35] used UDCA in 5 of 8 patients with liver fibrosis or secondary biliary cirrhosis confirmed via liver biopsy. Condat et al. [3] used UDCA in 3 of 4 patients with cholestasis who underwent endoscopic sphincterotomy and reported no recurrence of symptoms while the patients were receiving the treatment. Llop et al. [24] used UDCA in 10 of 14 patients with symptomatic PCC, including 5 patients with abdominal pain and cholestasis who were treated with UDCA alone, 2 patients with stricture but no calculi, and 3 of 6 patients with common bile duct stones that developed after sphincterotomy and ductal clearance. They reported “disappearance of symptoms and improvement of liver test results” during the follow-up of all treated patients. However, in the absence of appropriate controls, it is difficult to be sure that the achieved benefit is due to the use of UDCA.

The development of cholecystitis due to metallic stent use is a known complication. However, in our study, the cholecystitis rate was found to be higher after metallic stent compared to the literature rates. The most important reason for this is that since the stenosis reaches the main hepatic duct in all patients, long metallic stents must be used, thus increasing the possibility of closure of the cystic duct orifice. In addition, we speculate that in addition to the pressure of the metallic stent on the strictured bile ducts, there may also be stricture in the cystic duct secondary to PCC. However, in most patients, cholecystitis appears to regress with conservative treatment.

When the literature is examined, it is seen that metallic stents contribute to stricture resolution more effectively than plastic stents in the treatment of benign biliary stricture [36–38]. In a prospective study conducted by Tringali et al. [39] with 18 patients, it was observed that better results were obtained with stents applied for a longer period. We speculate that with the application of FCSEMS for a longer period of time, the chance of remodeling in the biliary tract increases and, accordingly, the success of the treatment increases.

This study had some limitations. First, the number of patients included was quite low. In addition, this study was an academic study wherein all the procedures were performed by an endoscopist who specializes in ERCP. Therefore, there is a need for multicenter studies involving endoscopists that are not ERCP experts to demonstrate the efficacy of FCSEMS placement. Moreover, prospective studies comparing plastic stents with FCSEMS can be more helpful in providing insight into treatment efficacy.

## Conclusion

FCSEMS placement is an effective method for treating biliary strictures in the presence of PCC. The use of FCSEMS eliminates the need for performing repeated endoscopic interventions during PCC treatment. Although acute cholecystitis is frequently observed after FCSEMS use, majority of the patients respond to conventional medical treatment. Patients should be followed up to monitor the recurrence of biliary strictures. Longer FCSEMS indwelling time may be beneficial for patients with recurrence.

## Data Availability

The datasets used and/or analyzed during the current study available from the corresponding author on reasonable request.
